# Supplementary material for: Effect of Light Conditions, Trichoderma Fungi and Food Polymers on Growth and Profile of Biologically Active Compounds in Thymus vulgaris and Thymus serpyllum
Source: Int J Mol Sci. 2024 Apr 29;25(9):4846. doi: 10.3390/ijms25094846 (PMC11084565; doi:10.3390/ijms25094846)
Supplement: Supplementary file 1 [file ijms-25-04846-s001.zip › ijms-2933826-supplementary.pdf]

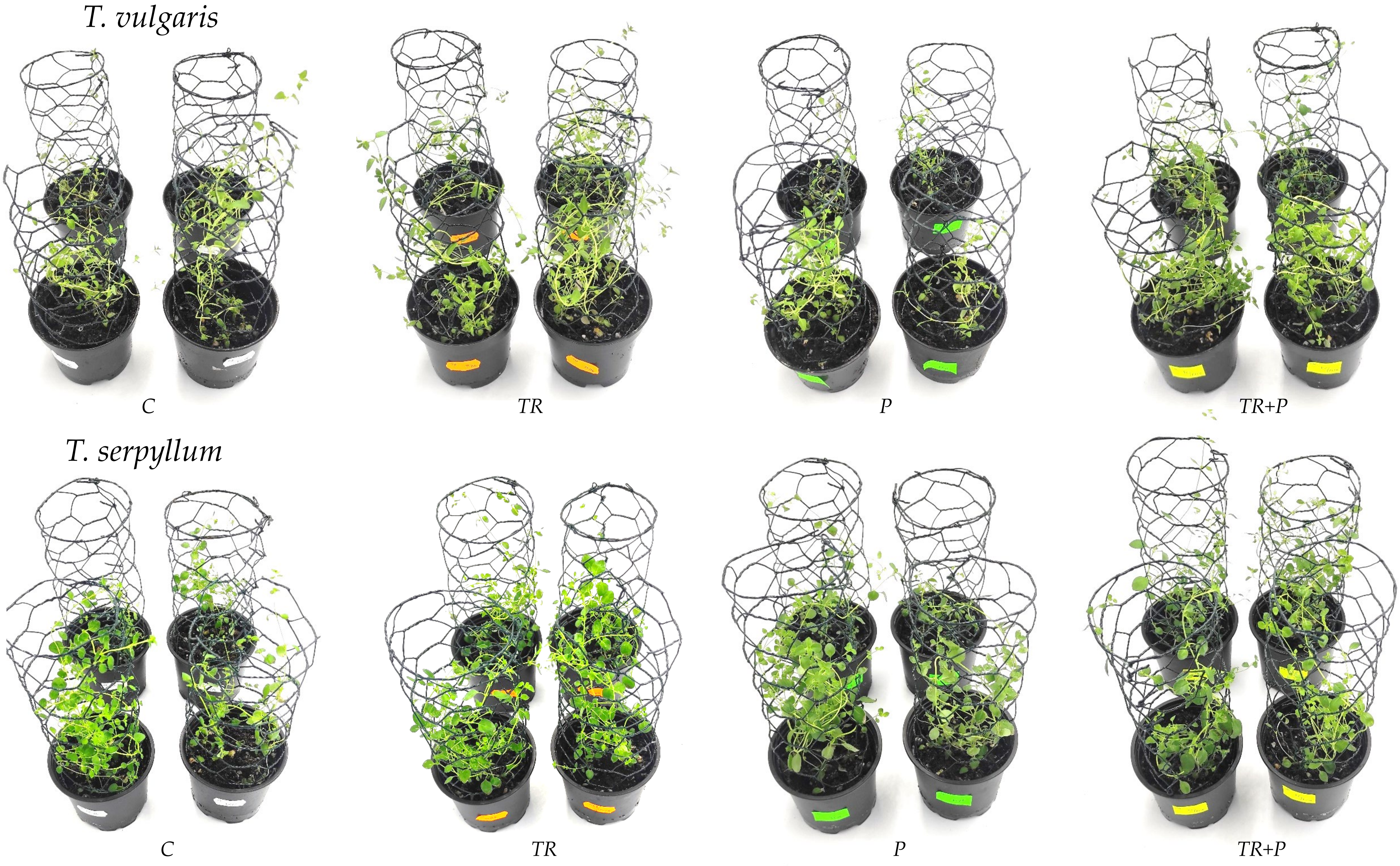

Figure S1. *T. vulgaris* and T. *serpyllum* plants cultivated in the semi-controlled conditions. Abbreviations: C, control plants, TR, plants grown in the soil supplemented with *Trichoderma*, P, plants grown in the soil supplemented with food polymers, TR+P, plants grown in the soil supplemented with *Trichoderma* and food polymers.

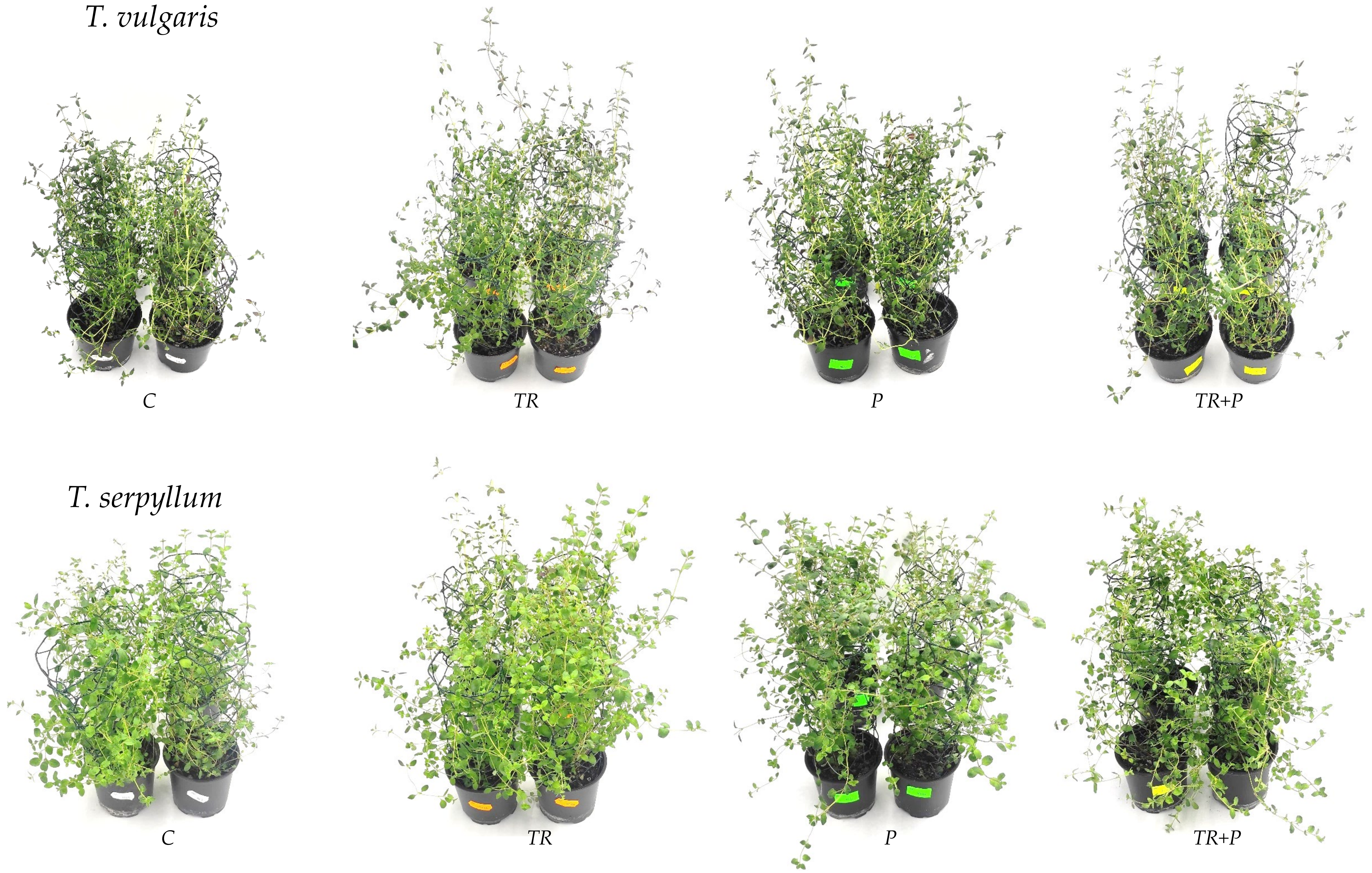

Figure S2. *T. vulgaris* and T. *serpyllum* plants cultivated in the controlled conditions, with lighting 174 $\mu mol \cdot m^{-2}\ s^{-1}$. Abbreviations: C, control plants, TR, plants grown in the soil supplemented with *Trichoderma*, P, plants grown in the soil supplemented with food polymers, TR+P, plants grown in the soil supplemented with *Trichoderma* and food polymers.

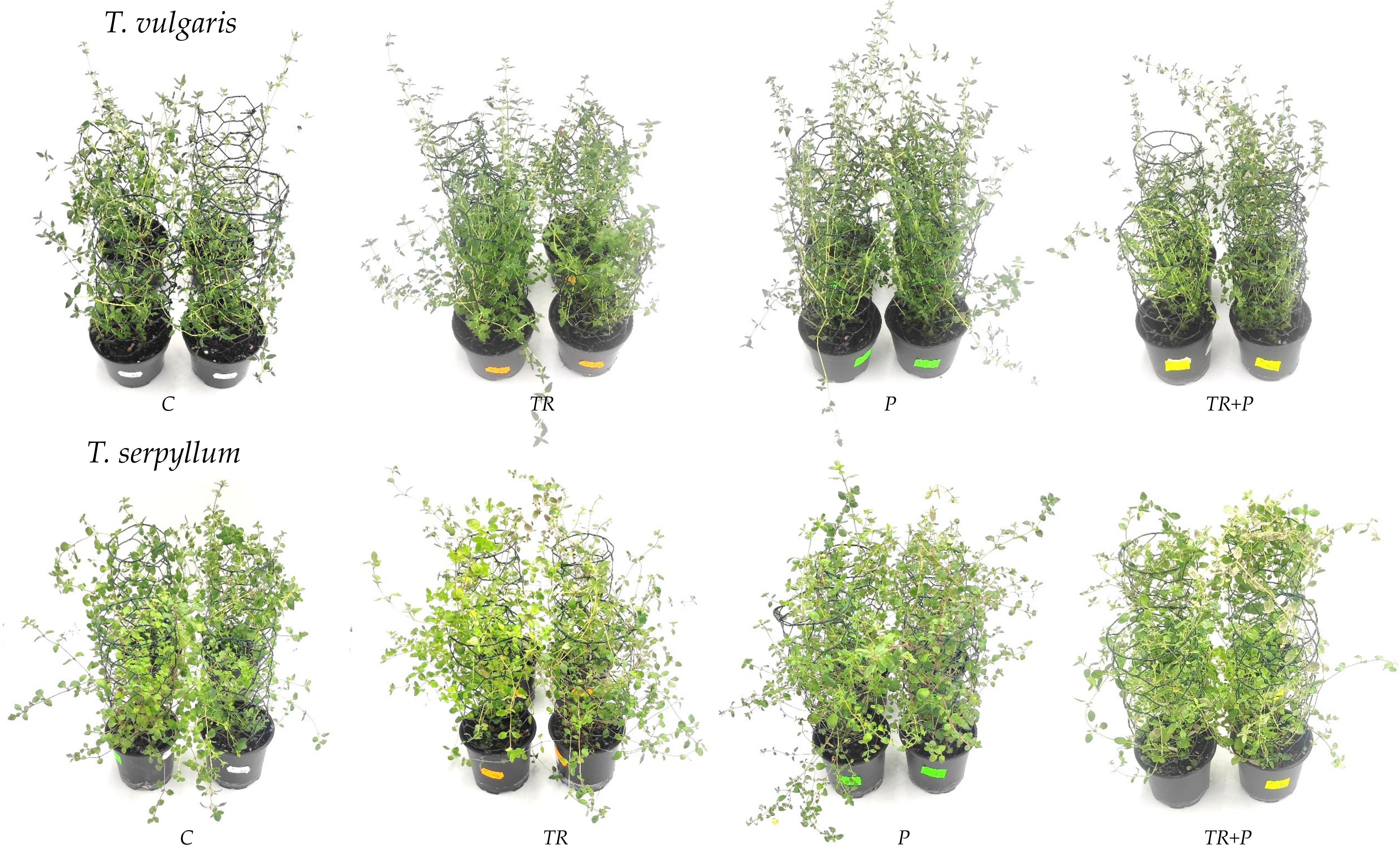

Figure S3. *T. vulgaris* and T. *serpyllum* plants cultivated in the controlled conditions, with lighting 600 μmol·m^{-2} s^{-1}. Abbreviations: C, control plants, TR, plants grown in the soil supplemented with *Trichoderma*, P, plants grown in the soil supplemented with food polymers, TR+P, plants grown in the soil supplemented with *Trichoderma* and food polymers.

Table S1. Results of a two-way ANOVA investigating the effects of the treatments studied on the tested parameters of *T. vulgaris* (a) and *T. serpyllum* (b) plants. Significance was indicated by an asterisk as follows (* $p < 0.05$; ** $p < 0.01$; *** $p < 0.001$).

(a)

**Dependent variable**	***F* value and significance**		
	**Lighting (L; df = 2)**	**Soil treatment (T; df = 3)**	**L x T (df = 6)**
Shoot biomass/FW [g]	59.08 ***	1.44	1.82
Length of shoot [cm]	10.60 ***	0.76	2.60 *
Content of chlorophyll a [mg g^{-1} FW]	1.32	0.87	2.62 *
Content of chlorophyll b [mg g^{-1} FW]	0.05	0.74	3.48 *
Content of carotenoids [mg g^{-1} FW]	18.91 ***	0.44	2.81 *
Content of anthocyanins [mg g^{-1} FW]	30.58 ***	0.17	2.77 *
Content of phenolics [mg g^{-1} FW]	409.37 ***	9.93 ***	5.80 ***
Content of flavonoids [mg g^{-1} FW]	44.15 ***	5.42 **	8.73 ***
Total antioxidant capacity [µM g^{-1} FW]	297.35 ***	20.96 ***	13.38 ***
Content of terpenoids [mg g^{-1} FW]	7.06 ***	9.81 ***	6.78 ***

(b)

**Dependent variable**	***F* value and significance**		
	**Lighting (L; df = 2)**	**Soil treatment (T; df = 3)**	**L x T (df = 6)**
Shoot biomass/FW [g]	32.62 ***	2.39 *	0.76
Length of shoot [cm]	43.74 ***	2.76 **	1.16
Content of chlorophyll a [mg g^{-1} FW]	48.08 ***	4.99 **	3.87 **
Content of chlorophyll b [mg g^{-1} FW]	18.35 ***	1.21 *	1.49 *
Content of carotenoids [mg g^{-1} FW]	0.79	0.34	1.81
Content of anthocyanins [mg g^{-1} FW]	2.74 *	1.75	1.27
Content of phenolics [mg g^{-1} FW]	172.87 ***	5.03 **	2.90 *
Content of flavonoids [mg g^{-1} FW]	88.37 ***	11.73 **	6.15 ***
Total antioxidant capacity [µM g^{-1} FW]	767.16 ***	16.60 **	5.49 ***
Content of terpenoids [mg g^{-1} FW]	11.81 ***	2.85 *	7.52 ***
